# Measuring Discrimination Against Transgender People at the University of the Basque Country and in a Non-University Sample in Spain

**DOI:** 10.3390/ijerph17072374

**Published:** 2020-03-31

**Authors:** Naiara Ozamiz-Etxebarria, Maitane Picaza, Eneritz Jiménez-Etxebarria, Jeffrey H. D. Cornelius-White

**Affiliations:** 1 Department of Developmental and Educational Psychology, University of the Basque Country, 48940 Leioa, Spain; eneritz.jimenez@ehu.eus; 2 Department of Didactics and School Organization, University of the Basque Country, 48940 Leioa, Spain; maitane.picaza@ehu.eus; 3 Department of Counseling, Leadership and Special Education, Missouri State University, Springfield, MO 65802, USA; jcornelius-white@missouristate.edu

**Keywords:** discrimination, transgender, university, education

## Abstract

Transgender people suffer from others’ negative attitudes in many situations. The university context is one environment where further progress has to be made to ensure the inclusion of transgender people. In this study, a sample of 376 undergraduate students was collected and their attitudes towards transgender people were analyzed. A comparison was made between number of years in university, and a sample from the general public. In addition, comparisons were made by gender, since the literature shows more negative attitudes toward transgender people in men than in women. The results show relatively positive attitudes toward transgender people among higher education students, but they have little knowledge of transgender identity. In turn, researchers found significant differences between different years in the university and between genders. These results support the need to expand knowledge about transgender people in the university environment.

## 1. Introduction

Transgender identity is an umbrella term that refers to a person who feels and lives as the opposite of the gender associated with the sex they were assigned at birth (transgender men and transgender women). The term transgender may refer to a wide range of social identities and gender presentations [1].

In a U.S. study, transgender people were classified into three groups: people who were assigned as men at birth and felt themselves to be women, people who were assigned as women at birth and felt themselves to be men, and finally, those who did not identify as either men or women [2]. In the last decade in particular, there has been growing evidence that there is in fact a considerable group of people who do identify with a non-binary gender identity [3].

Transphobia refers to negative beliefs and attitudes toward transgender people, including aversion and irrational fear of male women, female men, transvestites, transgender, or transsexual people [4]. Most of the research that has been done on gender discrimination has contributed by measuring and analyzing sexism and homophobia; in contrast, less research has been done on prejudice against people with transgender identities [5].

In the study by Factor et al. [2], they compared transgender people to their non-transgender brothers and sisters and found that transgender groups experienced significantly less social support from their families. In addition, they also experienced more harassment and discrimination than their non-transgender brothers and sisters. Another study by Lombardi et al. [6] investigated the prevalence of transgender people who had experienced violence and discrimination. They found that 60 percent of respondents reported being harassed on the street by strangers, through verbal abuse, assault with a weapon, and/or sexual assault. More than one-third (37%) also reported experiencing economic problems from discrimination through dismissal, demotion, or unfair treatment in the workplace [4].

Any social and cultural context should contribute to the inclusion of individuals based on respect and recognition of their rights, regardless of gender or other identity characteristics. On the other hand, too often the prevailing culture facilitates the creation of negative prejudices towards various groups. Both the connotations and the very language used to refer to people, as well as the social policies developed in a community, among other practices, can contribute to the stigmatization of people; this occurs when the plurality existing outside the woman or man binomial is not accepted [7]. An example of the negative conceptualization of transgender identity can be found in its description and classification in the Diagnostic and Statistical Manual of Mental Disorders (DSM) [8]. Despite the successive changes or corrections made in each edition, non-binary gender identity continues to be treated in a pathological prism that does not contribute to the normalization or well-being of transgender people [7]. In a qualitative study of attitudes of the social work students towards transsexual people, transsexuality was found to have a heterogeneity of negative meanings and implications. First, the identity of transsexuality was viewed as a treatable disease. Likewise, the students communicated the opinion that transgender people are not trained to raise families, and therefore should not have children. Also, some students communicated a stereotyped vision about the professions of transsexual people, identifying prostitution, hospitality, entertainment, and commerce as their main sources of employment [9].

On the other hand, despite the evidence of negative attitudes and transphobia experienced by transgender people on numerous occasions, studies have also found positive attitudes towards transgender people in various groups. For example, a study carried out with 225 health professionals found that they were a group with generally positive attitudes towards transgender people [10]. Another context in which positive attitudes toward transgender people were found was in feminist communities [11]. A more recent study showed that health professionals maintain generally favorable attitudes toward transgender people. In addition, gender differences in attitudes were found, with women showing more accepting attitudes toward transgender people than men [12].

In a Swedish general population study of attitudes toward transgender people with 668 people, results showed that a majority supported the possibility of transgender people undergoing sex reassignment; however, 63% thought that they should assume the costs. In addition, a majority supported the right to marry in their new sex, and to work with children. The right of transgender people to adopt and raise children was supported by 43%, while 41% opposed raising children as a right. The results indicated that those who believe that transsexuality is caused by biological factors have a less restrictive view of transgenderism than people who adopt a psychological view. Also, men and the older group were found to have a more restrictive view than women and the younger group. As with the previous study [12], a gender difference was found: men were less tolerant of transgender people than women [13]. In a qualitative study in Mexico between university and pre-university population, it was found that sex and educational level showed significant differences, both regarding the definition of the concept of transsexuality and in social attitudes toward sexual diversity (e.g., paternity/maternity, marriage, prohibition, and ownership of rights of lesbian, gay, transgender/transsexual, bisexual, and intersex (LGTBI) people, and affective expression in public). Thus, it is observed that myths and stereotypes have a smaller impact regarding transgender identity in women and pre-university students. On the other hand, the most negative attitudes were registered in men and university students [14,15].

In Spain, several research projects on transsexuality have been carried out, thanks to various contributions from activism and academia [16,17]. Within the educational framework, different studies confirm the gap that exists, even though it is an issue that matters to different groups that work with transgender people. For example, in medicine, where the framework for interpreting transsexuality comes from, the National LGBT Health Education Center offers educational programs, resources, and consultations to health care organizations, with the goal of optimizing quality and cost-effective health care for lesbian, gay, bisexual, and transgender (LGBT) people [18].

Another impetus for the visibility of transgendered children is the emergence of the Association of Families of Transgendered Children, Chrysalis, which is fighting for society, health care professions, and schools to address the needs of transgendered children on an equal basis with cisgender children. To this end, they have drawn up a list of some seventy schools that they call “trans friendly” to make it easier for children to survive and belong. Among the educational needs of the minors, the association points out that it is essential to “train all the personnel related to the educational process, the teachers, guidance counsellors, psychologists, assistants, social workers and management teams, as well as the training of the students” [19].

These examples reflect the social change that is currently taking place with respect to the interpretation of gender and sexuality. However, much remains to be done to enable these people to freely develop their identities. Various studies point out that, in the area of formal education, there are no training programs, and gender diversity is a subject that is not considered when different studies detect the need to work with students. For example, the European Union [20] elaborated the largest set of empirical information with the LGTB collective to date, with 93,000 people over 18 years old in the EU, where it was highlighted that the members of this community cannot be “themselves” in their daily lives. The results showed the following data: 47% of the respondents had felt discriminated against or harassed because of their sexual orientation or gender identity; more than 80% remembered negative comments or acts of bullying in the school environment; and 67% of the respondents declared that they hid their sexual orientation or gender identity during the school stage.

In Spain, homophobic bullying has always been present in schools. In 2011, INJUVE (Instituto Nacional de Juventud) highlighted that the homophobic group imposes its law in the classroom in front of the passivity of the rest of the students and teachers. In this line, some authors highlight the importance of the role of the observer as a facilitator of abuse [21,22]. A little later, in 2012/13, the Education Commission of the Madrid Lesbian, Gay, Transsexual and Bisexual Association (COGAM), together with the State Lesbian Federation, Gays, Transsexuals and Bisexuals (FELGTB), conducted a study on sexual diversity in the classrooms where they found that out of 653 children under 25 years of age who admitted to having suffered from school harassment because of their sexual orientation, 43% had even thought of suicide, evidencing the failure of the school system [23]. Against this background [24], they decided to focus on teachers’ attitudes and practices towards sexual diversity. The results show that infant, primary, and secondary school teachers think that not being heterosexual, or not conforming to gender expectations, are the reasons that generate more insults or rejection. Gender is also a variable where men are more likely to insult and less likely to ask for help, and women are more likely to address issues of diversity and coexistence in the classroom. Finally, there is a demand for constant training by the actors involved for both teachers and students, since both groups are victims of discrimination (insults, mockery, and exclusion) due to their personal characteristics [24].

In the Basque Country, The Department of Education, Language Policy and Culture has a master plan for co-education and prevention of gender violence. It works in collaboration with Berdindu, which is the Basque Government’s information and assistance service. In Berdindu, they work with different LGBTQ associations and the group called Berdindu Eskolak, for schools and families. However, although there is a commission in Bizkaia to coordinate the Provincial Government of Bizkaia, the City Council of Bilbao, the university, and the LGBTQ associations, the centers that have started to work are mainly primary and secondary schools. Furthermore, there is no specific protocol for the prevention and detection of homophobic and transphobic bullying [25].

In this path of discrimination prior to the university stage, schools do not guarantee measures against the stigmatization and marginalization of these people where the educational dimension of heterosexual and patriarchal norms continues [26,27]. In the universities, the panorama is not better either, the forms of identity and the new considerations associated with the inclusion of sexual diversity continue to be a pending subject, due to the strong cultural roots and gender binarism. Moreover, the concept of the university is historical and maintains its essence, and its raison d’être transcends all times, places, and social circumstances without the prevalence of reforms [28]. Proof of this is that despite the fact that different media, such as literature, cinema, plastic and audiovisual arts, or advertising, have introduced transsexual experiences into the educational sphere, the same does not occur in the academic sphere, where there is widespread misinformation about the LGTBI+ world [29].

Basque law 9/2019, of June 27 [30], includes in its articles 16 and 17 the obligation to incorporate methods, curricula, and educational resources that serve to increase understanding and respect for the diversity of gender identities, by dictating actions on transsexuality. However, this law only applies to basic education, leaving out universities.

Therefore, LGBT people continue to be constructed as minorities, with respect to a community of equals made up of heterosexual people. This leads them to be conceptualized from the discourse of otherness, and from a hegemonic and heteronormative position. This generates that the educational intervention reproduces discourses that consider these people as deficient, limiting them in agency [31].

Faced with this situation, trans people are self-excluded, and when it comes to choosing university studies, they opt for educational spaces perceived as safer and more respectful (such as studies related to art, teaching, nursing, or humanities, as well as those related to social change) instead of engineering or scientific/technical degrees, which are perceived as less desirable. Despite the existence of LGBT student associations in some universities, in general the university is created as an androcentric and Eurocentric space that is devoid of affectivity and focused on science. As a result, the university has been a space full of physical, bureaucratic, and symbolic barriers for LGBT people [32].

In Basque Country, like the rest of the world, the transgender group has historically been a marginalized group, and although today they are more accepted by society, attitudes of respect or normalization have yet to be promoted. One of the aspects to be developed is the provision of information in relation to transgender people, in fact, it has been found that health professionals (medicine, psychology, social education) do not perceive themselves to be qualified to provide care services to transgender people, and may therefore exhibit avoidance behaviors [10]. Therefore, it is important to ensure that professionals who are in contact with transgender people are familiar with the history and culture of this population, to facilitate understanding and good practices.

The University of the Basque Country (UPV/EHU) is located in the Basque region of northern Spain. Distributed on three campuses with many clusters (one campus in each of the historical territories of the current Basque Autonomous Community) which group together 20 faculties and schools, the UPV/EHU makes a decisive contribution to the reality of the Basque Country, to the extent that it would be inconceivable today without the daily contribution of this institution, and without the rich and intense intellectual debate that is generated around it. 

The first objective of the research is to analyze the level of knowledge and attitudes towards transgender people at different levels of the University of the Basque Country (UPV/EHU) and in non-university or a general public sample collected outside the university. The second objective is to measure the comparison between different degrees and general public sample in terms of knowledge and attitudes towards transgender people according to sex, and previous knowledge about the reality of transgender people. With all of this, the aim is to identify the training needs in relation to the reality of transgender people in the university environment, and to make a comparison with the results of previous scientific literature.

## 2. Materials and Methods

### 2.1. Participants

In the Basque Country, there were 41,123 people enrolled in university degree studies in the 2016/17 academic year (EUSTAT, 2019), of which 22,703 were women and 18,420 were men. According to data from the UPV/EHU (2017), the number of people enrolled in the university of the Basque Country (UPV/EHU) was 36,484. In the 2013/2014 academic year, the number of students enrolled in the UPV/EHU was 33,730; 22,703 were women, and 18,420 were men.

A convenience sample was taken among students of different degrees of the Public University of the Basque Country (UPV/EHU), specifically from the degrees of law, social education, history, engineering, primary education, medicine, and psychology. A sample of the general public was also collected from outside the University. The researchers offered voluntary participation in this study to the students, and the final participation was 376 persons. The average age was 21.93. Of those who participated, 63.8% (*n* = 240) were women, 31.1% (*n* = 117) were men, and 5.1% (*n* = 19) identified as other, that is, were not identified as either men or women. The very response rate of women to men is interesting in itself, given that one would expect a sample difference of only 10%, rather than 33%, if a representative sample was used.

All persons participated voluntarily, received information about the research procedure, and gave consent before participating in the study. The procedure followed was approved by the Ethics Committee with respect to the Declaration of Helsinki of the World Medical Association.

### 2.2. Gender and Transphobia Scale

The Gender and Transphobia Scale is a scale developed and validated in Canada (GTS; Hill and Willoughby, 2005) that analyzes negative attitudes toward trans people. It assesses the cognitive (gender), affective (transphobia), and behavioral (gender attack) components of negative attitudes. It is a scale that has been translated and validated in several cultures. For this study, the short version of the Gender and Transphobia Scale validated in Spanish has been used, with a stable factorial structure and adequate reliability (Carrera-Fernández et al., 2013). It is a test with good psychometric properties, and the shorter length of the instrument means a shorter passage time and a possible increase in the effectiveness of the evaluation processes.

The scale consists of 12 items that measure the variables of gender abuse (gender bashing), transphobia, and sexism (genderism). Sexism is a belief system based on a heteronormative social model. Sexism devalues people who do not conform to their gender roles, or whose sex is not consistent with their gender. Transphobia is the attitudinal component; it includes negative feelings, aversion, and fear of people who transgress the rigid two-gender model. Gender abuse is the act of victimizing a person emotionally, physically, sexually, or verbally for being transgendered; it is the behavioral component of sexism [5].

The first six items of the short version of the GTS measure gender abuse, and the last six measure transphobia and sexism. Responses are answered on a Likert scale from 1 to 7 with the values 1 = strongly agree; 2 = agree; 3 = somewhat agree; 4 = neutral; 5 = somewhat disagree; 6 = disagree; and 7 = strongly disagree. Lower scores indicate a higher level of transphobic attitudes. For the factors gender abuse and sexism/transphobia, the lowest score that can be obtained is a 7, indicating high levels of gender abuse and transphobia/sexism. The highest score that can be obtained on these two factors would be 42, indicating the absence of gender abuse and transphobia/sexism.

### 2.3. Other Variables Measured in This Study

Using a Likert scale from 1 to 10, participants have self-assessed their knowledge of transgender identity. On this scale, a 1 corresponds to no knowledge about the subject, and a 10 represents optimal knowledge about the subject.

Other variables collected were (b) age, (c) gender, and also (d) whether they know any person who is transgender.

### 2.4. Procedure

This project was approved on October 31, 2019 by the Ethics Committee of the University of the Basque Country CEISH-UPV/EHU, BOPV 32, 17/2/2014, with code M10_2019_223. The research project was titled “Attitudes and Beliefs About Gender and Transgender in University Students”.

In order to carry out the research, the project was presented to the Comité de Ética para las Investigaciones relacionadas con Seres Humanos (CEISH) Ethics Committee of the University of the Basque Country UPV/EHU, in order to respect the established principles of the Declaration of Helsinki. After obtaining permission, the research team contacted different teachers in the fields of law, social education, history, engineering, primary education, medicine, and psychology, who gave their approval to carry out this study.

The researchers approached the faculties of the mentioned degrees and informed the students about the procedure of the study. The people who decided to participate voluntarily in the study filled out the instruments that were provided to them through the Google Forms platform. In turn, the sample from the general public participated in the study by means of virtual boards on which the need for volunteers for this study was announced.

### 2.5. Statistical Analysis

The data were collected through Google Forms and analyzed in the city of Bilbao (Spain) with SPSS version 25, IBM SPSS. Sample data were described using means, standard deviations, and percentages. A Multivariate analysis of variance (MANOVA) was performed for the two factors of the short version of the Gender and Transphobia Scale, according to the subsamples from which they came (academic major or non-university). A MANOVA was also run to compare the two factors and the two genders with sufficient sample size for comparisons (i.e., male and female). Because the Box and Levene’s tests of homogeneity of variance were significant on both MANOVAs, showing a violation of that assumption, the researchers employed Pillai’s trace as the multivariate test. Bonferroni corrections were used to create more stringent standards for significance in differences between the means, to account for the multiple tests run. Results are provided in tables.

## 3. Results

Descriptive results are presented first. Table 1 shows the sociodemographic data of the sample.

The descriptive results on the Gender Bashing and Transphobia/Sexism scales by gender are presented in Table 2. The third group of gender (others) was too small to make a comparison in the inferential analyses.

As for the participants’ academic majors in the study, 29.8% were medical students, 13.8% primary school students, 14.1% engineering students, 17.8% social education students, 5.6% law students, 2.9% history students, 2.6% psychology students, and 13.4% were general public. To address the issue of small sample sizes in some academic majors, the researchers considered combining those with under 20 participants (law, history, and psychology) to form a larger group. These three academic majors reflect similar academic content, and the scores were not significantly different from one another on either gender bashing or transphobia/sexism, according to two ANOVAs that were run. Therefore, they were combined for an adequate size group, referred to as law–history–psychology at 11.1% of the sample.

The inferential results are presented next. Results show that there were significant gender differences, *F* (1.355) = 6.91, *p* < 0.001, Pillai’s trace = 0.17, *η_p_^2^* = 0.17. Specifically, there were differences on the Gender Bashing, *F* (1.355) = 39.43, *p* < 0.001, *η_p_^2^* = 0.10 and Transphobia/ Sexism, *F* (1.355) = 43.54, *p* < 0.001, *η_p_^2^* = 0.11 scales. Men demonstrated more negative attitudes than women.

Results show that there were significant differences based on academic major or the general public, *F* (8.704) 6.89, *p* < 0.001, Pillai’s trace = 0.15, *η_p_^2^* = 0.07. Specifically, there were differences on the Gender Bashing, *F* (4.352) = 5.07, *p* < 0.001, *η_p_^2^* = 0.06, and Transphobia/ Sexism, *F* (4.352) = 11.01, *p* < 0.001, *η_p_^2^* = 0.11. Engineering students demonstrated more gender bashing attitudes than medicine or law–history–psychology students, and more transphobia/sexism than all other academic majors, but not the general public (See Table 3). Bonferroni corrections called for setting the significance at *p* < 0.01.

## 4. Discussion

The plurality of the participants (29.7%) were medicine students. These data indicate that medical students have shown greater interest in the study, either because of awareness of transgender identity, or because it is a profile of students with a high level of participation in academic tasks. In fact, studies have highlighted the positive attitudes that health professionals have toward transgender people [10,12]. Overall, participants on average viewed themselves as having a moderate amount of knowledge about transgender people, and a majority knew a transgender person.

In terms of the difference in means for gender abuse and transphobia/sexism, there are significant differences between men, women, and non-binary people. Women are the people who show more positive attitudes in both factors, and men are the people with more negative attitudes. These results are consistent with research, where a higher proportion of negative attitudes were found in men than in women, including a Mexican study, in which worse attitudes were observed in men with a higher educational level, and stereotyped ideas were detected [9,13,14,15].

As for the different academic majors and the general public, engineering students demonstrated significantly more transphobia/sexism than the other academic majors, and more gender bashing than the medicine and the law-history-psychology groups. Nevertheless, it must be taken into account that overall, the attitudes were generally positive in all groups, including the non-university sample. This may be due to the fact that the people who have decided to participate in this study may have been more aware or accepting of transgender people than people who did not choose to participate.

These data coincide with the study by Pichardo et al. [16], which states that feminized studies and humanities, and social change-related studies, are perceived as safer and more respectful of transgender people, and traditionally masculinized degrees, such as engineering and scientific/technical, are perceived as less safe.

As for the comparison of the general public sample with different university majors, no significant differences were seen. If the university environment were to promote gender and transgender training and facilitate the normalization of transgender people, perhaps the influence of training would result in a greater proportion of positive attitudes on the part of students in the process of training, and, therefore, result in greater respect and awareness. However, our data confirm the statements of Castro and Esver [29], who claim that there is widespread misinformation about the LGTBI community in the university environment.

Finally, it should be stressed that discrimination against LGTBI people occurs in the educational community in general, and also in other spaces of the social community, like family or the workplace, as well as the fact that receiving unfavorable treatment from the public has the tendency to result in suicidal attempts [23]. That is why it is important that the present study not only measured the perspective from the university students, but also the general public, because promoting society’s knowledge and understanding toward LGTBI is also crucial.

## 5. Conclusions

This study has been carried out on a sample of the Basque Autonomous Community. The findings of this study confirm claims from previous research regarding the limited knowledge of transgender identity. Even so, some differences have been found with respect to previous studies, in terms of health professionals and women having more positive attitudes than the general public and men. In this study, engineering students were found to have more negative attitudes than other academic majors. The research supports the need to increase knowledge about transgender identity in the university system. In addition, since transgender people may opt for humanities degrees rather than engineering degrees, it would be important to increase knowledge and awareness of transgender people among students not so closely related to the humanities. This would help the inclusion of transgender people or the LGTBI collective in such an environment that has been, until now, hostile.

It is necessary to continue obtaining more scientific evidence in this area and to compare the results with larger sample sizes, particularly in some academic majors with small numbers of participants. It would also be relevant to set the same objectives in a sample of different age ranges and carry out an intergenerational comparison, since the new generations seem to have more positive attitudes towards transgender people. Finally, the need has been detected to develop evaluation instruments in Spanish for attitudes towards transgender people. For this reason, the present research team has translated, and is in the process of validating, the Transgender Attitudes and Beliefs Scale (TABS) test together with its authors Kanamori, Cornelius-White, Pegors, Daniel, and Hulgus from Missouri State University. It is hoped that by 2020, these scales will be validated and can be used in future research.

## Figures and Tables

**Table 1:** Means, standard deviations, and percentages of the sample in terms of age, gender, knowing a transgender person, and knowledge of the transgender topic.

Age	Mean	SD	Max			Min	
	21.9	11.5	78			17	
Gender	**Women**		**Men**			**Other**	
	N	%	N		%	N	%
	240	63.8	117		31.1	19	5.1
Knowing a transgender person	**No**		**Yes, But Not Personally**			**Yes, Personally**	
	N	%	N		%	N	%
	139	36.9	189		50.3	48	12.8
Knowledge on the transgender topic from 1 to 10	**Less Than 3**		**Between 3 And 7**			**More Than 8**	
	N	%	N		%	N	%
	6	1.6	233		61.9	137	36.5
Knowledge on the transgender topic from 1 to 10	**Mean**			**SD**			
	6.7			1.8			

**Table 2:** Means and standard deviations for the three gender categories.

Attitude Scale	Mean (SD) Male	Mean (SD) Female	Mean (SD) Other
Gender bashing	38.93 (3.84)	40.93 (2.38)	40.26 (2.51)
Transphobia/ Sexism	36.69 (6.81)	40.32 (3.58)	39.89 (3.73)

**Table 3:** MANOVA results comparing means on Gender Bashing and Transphobia/ Sexism, according to academic major.

Dependent Variable	Academic Major	Academic Major	Mean Difference	SE	*p*-Value
Gender Bashing	Engineering	Medicine	−2.06	0.48	<0.001
		Law-History-Psychology	−2.23	0.65	<0.006
Transphobia/Sexism	Engineering	Medicine	−3.52	0.78	<0.001
		Primary Education	−3.63	0.99	<0.002
		Law-History-Psychology	−4.99	1.06	<0.001
		Social Education	−5.72	0.9	<0.001
